# Schoolchildren with Learning Difficulties Have Low Iron Status and High Anemia Prevalence

**DOI:** 10.1155/2016/7357136

**Published:** 2016-09-15

**Authors:** F. P. N. Arcanjo, C. P. C. Arcanjo, P. R. Santos

**Affiliations:** ^1^Medical Faculty, Department of Master's Degree in Family Health, Federal University of Ceará, Sobral Campus, Av. Gerardo Rangel 100, 62.042-280 Sobral, CE, Brazil; ^2^Medical Faculty, University of Fortaleza, Fortaleza, CE, Brazil

## Abstract

*Background*. In developing countries there is high prevalence of iron deficiency anemia, which reduces cognitive performance, work performance, and endurance; it also causes learning difficulties and negative impact on development for infant population.* Methods*. The study concerns a case-control study; data was collected from an appropriate sample consisting of schoolchildren aged 8 years. The sample was divided into two subgroups: those with deficient initial reading skills (DIRS) (case) and those without (control). Blood samples were taken to analyze hemoglobin and serum ferritin levels. These results were then used to compare the two groups with Student's *t*-test. Association between DIRS and anemia was analyzed using odds ratio (OR).* Results*. Hemoglobin and serum ferritin levels of schoolchildren with DIRS were statistically lower when compared to those without, hemoglobin *p* = 0.02 and serum ferritin *p* = 0.04. DIRS was statistically associated with a risk of anemia with a weighted OR of 1.62.* Conclusions*. In this study, schoolchildren with DIRS had lower hemoglobin and serum ferritin levels when compared to those without.

## 1. Introduction

Iron deficiency anemia (IDA) is the most widespread preventable nutritional problem; data from the World Health Organization (WHO) estimates that more than 2 billion people suffer from IDA [1].

Iron deficiency (ID) and IDA have been studied in human and animal models as the cause of developmental abnormalities and there is already a consensus that adequate iron levels are necessary for normal neurodevelopment [2, 3].

Human studies have tried to correlate the effects of early ID to mental and cognitive development. Cohort studies undertaken by Lozoff and colleagues have reported an interesting relationship on such effects [4–7].

A recent census shows that Brazil has around 14.9 million illiterate people over the age of 10 years (7.8% of the total population); the percentage of schoolchildren failing a school year or abandoning school is 20.5% (national average), reaching up to 27.6% in poorer areas [8].

Although ID cannot be isolated as the sole problem causing deficient initial reading skills (DIRS), it should be considered as one of the elements involved in learning deficit. Facing this reality, investigations should be carried out to analyze and reduce the consequences of ID [9].

This study had the objective of evaluating whether children with DIRS in the 2nd year of fundamental schooling (8-year olds) had different iron status to those without DIRS and evaluating if there is an association between DIRS and anemia in these children.

## 2. Materials and Methodology

This study was conducted in the city of Sobral, in the northeast of Brazil. Fieldwork was done in August 2014. This study used a convenience sample of schoolchildren aged 8 years from one public school located in the urban zone (*n* = 105). Children enrolled in the study were examined by a qualified medical officer, and those with chronic disease or acute infections were excluded from the study and referred for treatment.

DIRS (in Sobral, Brazil) is defined as a child not being able to read/write up to the age of eight years; this learning difficulty is identified according to an internal instrument that assesses students in their native language (Portuguese) (unpublished material). In summary, this external assessment consists of the recording of a student's oral reading. During the assessment, all children are required to read the material presented (words, sentences, and a short text) in individual interview character; a writing assessment tool, applied in the form of dictation, also integrates this evaluation. Performance was assessed by qualified professionals from the Secretariat of Education and Development of the Municipality and classified as adequate or inadequate for age.

The study concerns a case-control study. Data was collected from a sample consisting of schoolchildren from the 2nd year of fundamental schooling at a public school. This group was divided into two subgroups: those with learning difficulties (case) and those without (control).

**Figure 1:**
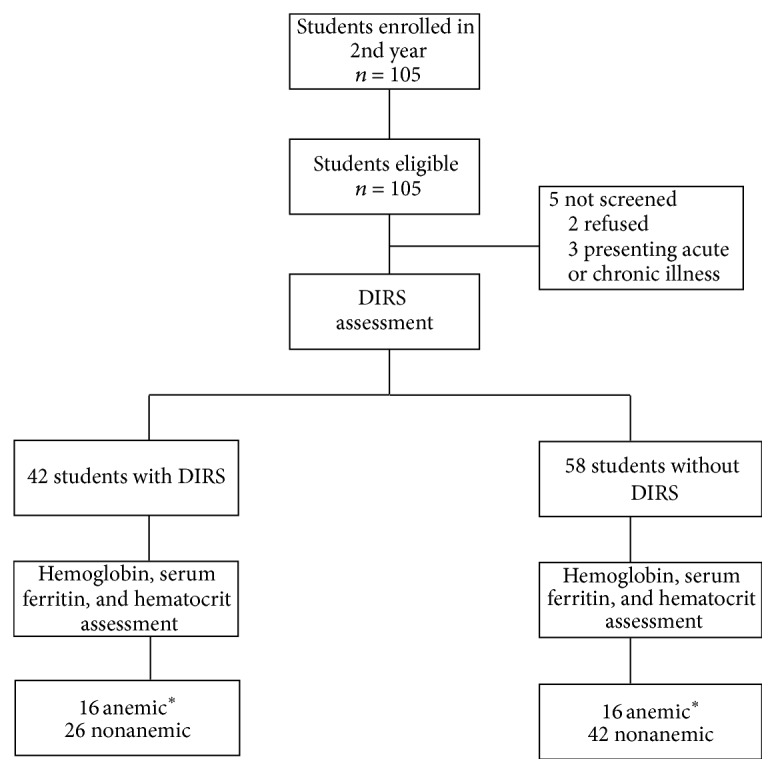
Study profile.  ^*∗*^Hemoglobin concentration <115 g/L.

Inclusion in the study was made as follows. All of the 105 students enrolled in the 2nd year were eligible (study population). These students were offered the opportunity to participate in the study, and participation was made official through the signing of a parental consent form. Five (4.76%) students were excluded at baseline before blood analysis: 2 (1.90%) refused to participate; 3 (2.86%) presented acute or chronic illness (Figure 1).

After enrollment in study, all students were assessed, on the same day, by local representatives from the Secretariat of Education. Blood samples were also collected from each student for hemoglobin, hematocrit, and serum ferritin analysis. Anemic children were referred for treatment after hemoglobin analysis.

The main outcomes of the study to be analyzed were hemoglobin, hematocrit, and ferritin serum levels. Cutoff for anemia as a hemoglobin value (Hb) in children aged 8 years was Hb < 115 g/L; the cutoff value of serum ferritin for the diagnosis of iron deficiency was <15 *µ*g/L [1].

Data was managed and analyzed using Epi Info version 6 [10]. Unpaired Student's *t*-tests were used to assess differences in mean hemoglobin, hematocrit, and ferritin serum levels between subgroups; *p* < 0.05 was used to define significant associations. Fisher's exact test was used to compare categorical variables. For the analysis of data, 2 × 2 contingency table was used and odds ratios (OR) were used to compare the relative odds of the occurrence of anemia.

The study was approved by the Ethics Committee for Research at State University “Vale do Acaraú” and developed integrally following ethical principles established by the National Health Council Resolution #466/2012.

## 3. Results

Of the 105 students eligible for participation in this study, 5 withdrew before enrollment (Figure 1). Hence, the study was conducted with 100 participants.  Table 1 displays the baseline characteristics of the sample. Forty-two out of 100 students presented learning difficulty (cases), while 58 did not (controls); mean age in the groups was 8.9 ± 0.6 and 9.0 ± 0.5 years (*p* = 0.77), respectively. The baseline characteristics of the study population available for this analysis (mother's schooling, family income, birth weight, and breast feeding), between the groups (case and control), did not differ. In summary, the general characteristics at baseline were quite similar in those aspects analyzed in both groups (Table 1).

Each group (case and control) presented 16 anemic participants. The prevalence ratio (prevalence of DIRS in children with anemia divided by the prevalence of DIRS in children without anemia) was 1.31.

In children with DIRS, mean hemoglobin, hematocrit, and serum ferritin were 118 ± 6.14 g/L, 35.7 ± 2.12%, and 28.8 ± 13.2 *µ*g/L, respectively, and in schoolchildren without DIRS 122 ± 9.68 g/L, 35.9 ± 3.09%, and 34.9 ± 15.6 *µ*g/L. The comparative analysis of data from the two groups resulted in the *p* values of 0.02 for hemoglobin, 0.69 for hematocrit, and 0.04 for serum ferritin (Table 2).

Anemia was significantly associated with an increased risk of DIRS with a weighted OR of 1.62.

## 4. Discussion

### 4.1. Main Findings of This Study

Mean hemoglobin and serum ferritin levels of schoolchildren with DIRS were statistically lower when compared to those without, *p* = 0.02 and *p* = 0.04, respectively, identifying an association between iron status and DIRS, with IDA increasing the risk of DIRS. DIRS was statistically associated with a risk of anemia with a weighted OR of 1.62.

### 4.2. What We Already Know

Extensive reviews have consistently observed associations between ID/IDA and deficits in cognitive or behavioral performance in children [11–17]. In general terms, studies have concluded that cognitive performance of children with ID/IDA tended to improve with iron treatment in children aged over 2 years; however, performance showed little or no improvement in children under the age of 2 years [4, 13, 16, 18–20].

However, the conclusions from some of these studies have been questioned due to problems related to limited statistical power, having a small number of participants, being of short-duration, and not being randomized or double-blind. Two significant RCTs had diverging results concerning improving performance deficit after treatment for anemia [21, 22].

In a systematic review, Sachdev et al. [12] concluded that iron supplementation modestly improves mental development scores. This improvement can be better seen in intelligence tests with previously anemic or iron-deficient children over the age of 7 years. Nevertheless, there is no substantial evidence to confirm that iron treatment is effective on mental or motor development in children under the age of 27 months.

Other studies have demonstrated lower scores on cognitive testing of iron-deficient anemic adolescents when compared to their nonanemic counterparts [23–26]. The latter, a large study involving a survey of 5,398 American children aged 6–16 years, concluded that children with ID (with or without anemia) had more than double the risk of scoring below average in the math test [24].

In 2001, WHO highlighted the importance of the early prevention of ID/IDA as a mode to prevent the long-term negative consequences of impaired mental development on the formation of unqualified human resources [1]. The prevalence of IDA in developing countries is extremely high ranging between 29.3 and 67.6% [27, 28]. ID in this period of significant brain plasticity may lead to possible sequels in cognition and learning for these children [3, 11, 15].

According to Radlowski and Johnson [17] ID delays learning and motor and emotional development; individuals exposed to ID during the perinatal period are at high risk for not reaching educational standards later in life.

Piñero and Connor [29] report that ID decreases iron concentration in the brain, leading to numerous behavioral symptoms such as irritability, apathy, reduced ability to concentrate, and other cognitive deficits.

### 4.3. What This Study Adds

Our study showed that children with DIRS had lower mean levels of Hb and serum ferritin when compared to those without DIRS. We also found a positive association between DIRS and anemia; anemia was significantly associated with an increased risk of DIRS with a weighted OR of 1.62.

### 4.4. Limitations of This Study

The cause and effect relationship between iron deficiency and DIRS is hard to measure through RCTs due to ethical problems that limit applicability. The use of case-control, cohorts, and cross-sectional studies and research with animal models denote the importance of adequate iron ingestion for human cognitive development.

## 5. Conclusions

Our study demonstrates that it is imperative to prevent ID in developmental periods of life when iron demands are higher, as socioemotional development is uniquely vulnerable to ID and IDA, and the effects of early ID may be irreversible. Developmental loss through ID and IDA can be avoided through adequate maternal iron status, prevention of premature birth, delayed cord clamping, and iron rich diets.

## Figures and Tables

**Table 1:** Baseline characteristics of sample, by group.

Baseline characteristics of sample	With learning difficulty (case)	Without learning difficulty (control)	*p* value

*N*	42	58	
Age (years; SD^*∗*^)	8.9 (0.6)	9.0 (0.5)	0.77^†^
Gender (male : female)	0.9 : 1	1 : 1	
Mother ≤ 8 y of schooling	30	40	0.82^‡^
Family's monthly income ≤ 300 USD	28	45	0.26^‡^
Birth weight (kg mean; SD^*∗*^)	3.1 (0.7)	3.0 (0.6)	0.88^†^
Birth weight < 2.5 kg	4	8	0.76^‡^
Breast-fed for any period of time	39	50	0.35^‡^
Breast-fed ≥ 6 m	16	24	0.84^‡^

All data are mean or number (%) unless indicated otherwise.

^*∗*^SD standard deviation.

^†^Unpaired *t*-test.

^‡^Fisher's exact test.

**Table 2:** Comparative analysis between children with DIRS and those without.

	With DIRS (*n* = 42)	Without DIRS (*n* = 58)	*p* value^‡^
	Case	Control	
Hemoglobin (g/L) ± SD^*∗*^	118 ± 6.14	122 ± 9.68	0.02
CI^†^	115.0, 120.1	119.3, 123.7	
Hematocrit (%) ± SD	35.7 ± 2.12	35.9 ± 3.09	0.69
CI	34.86, 36.53	35.20, 36.62	
Serum ferritin (*µ*g/L) ± SD	28.8 ± 13.2	34.9 ± 15.6	0.04
CI	24.27, 33.25	31.07, 38.72	

All data are mean.

^*∗*^SD = standard deviation.

^†^CI = 95% confidence interval.

^‡^Based on unpaired Student's *t*-tests.

## References

[1] World Health Organization (2001). *Iron Deficiency Anaemia, Assessment, Prevention and Control: A Guide for Program Managers*.

[2] Lozoff B., Georgieff M. K. (2006). Iron deficiency and brain development. *Seminars in Pediatric Neurology*.

[3] Beard J. L., Connor J. R. (2003). Iron status and neural functioning. *Annual Review of Nutrition*.

[4] Lozoff B., Brittenham G. M., Wolf A. W. (1987). Iron deficiency anemia and iron therapy effects on infant developmental test performance. *Pediatrics*.

[5] Lazoff B., Wolf A. W., Jimenez E. (1996). Iron-deficiency anemia and infant development: effects of extended oral iron therapy. *Journal of Pediatrics*.

[6] Lozoff B., Jimenez E., Wolf A. W. (1991). Long-term developmental outcome of infants with iron deficiency. *The New England Journal of Medicine*.

[7] Lozoff B., Jimenez E., Hagen J., Mollen E., Wolf A. W. (2000). Poorer behavioral and developmental outcome more than 10 years after treatment for iron deficiency in infancy. *Pediatrics*.

[8] Brasil (2006). *Ministério da Educação. Instituto Nacional de Estudos e Pesquisas Educacionais (INEP)*.

[9] Brasil Parâmetros curriculares nacionais: adaptações curriculares.

[10] Dean A. G., Dean J. A., Coulombier D. (1994). *Epi Info, Version 6: A Word Processing, Database, and Statistics Program for Epidemiology on Microcomputers*.

[11] Yager J. Y., Hartfield D. S. (2002). Neurologic manifestations of iron deficiency in childhood. *Pediatric Neurology*.

[12] Sachdev H. P. S., Gera T., Nestel P. (2005). Effect of iron supplementation on mental and motor development in children: systematic review of randomised controlled trials. *Public Health Nutrition*.

[13] Logan S., Martins S., Gilbert R. (2001). Iron therapy for improving psychomotor development and cognitive function in children under the age of three with iron deficiency anaemia. *Cochrane Database of Systematic Reviews*.

[14] McCann J. C., Ames B. N. (2007). An overview of evidence for a causal relation between iron deficiency during development and deficits in cognitive or behavioral function. *American Journal of Clinical Nutrition*.

[15] Lozoff B. (2007). Iron deficiency and child development. *Food and Nutrition Bulletin*.

[16] Grantham-McGregor S., Ani C. (2001). A review of studies on the effect of iron deficiency on cognitive development in children. *Journal of Nutrition*.

[17] Radlowski E. C., Johnson R. W. (2013). Perinatal iron deficiency and neurocognitive development. *Frontiers in Human Neuroscience*.

[18] Lozoff B., Brittenham G. M., Viteri F. E., Wolf A. W., Urrutia J. J. (1982). The effects of short-term oral iron therapy on developmental deficits in iron-deficient anemic infants. *The Journal of Pediatrics*.

[19] Walter T., De Andraca I., Chadud P., Perales C. G. (1989). Iron deficiency anemia: adverse effects on infant psychomotor development. *Pediatrics*.

[20] Seshadri S., Gopaldas T. (1989). Impact of iron supplementation on cognitive functions in preschool and school-aged children: the Indian experience. *The American Journal of Clinical Nutrition*.

[21] Idjradinata P., Pollitt E. (1993). Reversal of developmental delays in iron-deficient anaemic infants treated with iron. *The Lancet*.

[22] Aukett M. A., Parks Y. A., Scott P. H., Wharton B. A. (1986). Treatment with iron increases weight gain and psychomotor development. *Archives of Disease in Childhood*.

[23] Webb T. E., Oski F. A. (1973). Iron deficiency anemia and scholasticachievement in young adolescents. *The Journal of Pediatrics*.

[24] Halterman J. S., Kaczorowski J. M., Aligne C. A., Auinger P., Szilagyi P. G. (2001). Iron deficiency and cognitive achievement among school-aged children and adolescents in the United States. *Pediatrics*.

[25] Lukowski A. F., Koss M., Burden M. J. (2010). Iron deficiency in infancy and neurocognitive functioning at 19 years: evidence of long-term deficits in executive function and recognition memory. *Nutritional Neuroscience*.

[26] More S., Shivkumar V. B., Gangane N., Shende S. (2013). Effects of iron deficiency on cognitive function in school going adolescent females in rural area of central India. *Anemia*.

[27] Arcanjo F. P. N., Pinto V. P. T., Coelho M. R., Amâncio O. M. S., Magalhães S. M. M. (2008). Anemia reduction in preschool children with the addition of low doses of iron to school meals. *Journal of Tropical Pediatrics*.

[28] Benoist B., Mclean E., Egli I., Cogswell M. (2008). *Worldwide Prevalence of Anemia 1993–2005*.

[29] Piñero D. J., Connor J. R. (2000). Iron in the brain: an important contributor in normal and diseased states. *Neuroscientist*.

